# Metapath Aggregated Graph Neural Network and Tripartite Heterogeneous Networks for Microbe-Disease Prediction

**DOI:** 10.3389/fmicb.2022.919380

**Published:** 2022-05-31

**Authors:** Yali Chen, Xiujuan Lei

**Affiliations:** School of Computer Science, Shaanxi Normal University, Xi’an, China

**Keywords:** microbe-disease associations, heterogeneous network, metapath aggregated graph neural network, multi-head attention mechanism, COVID-19

## Abstract

More and more studies have shown that understanding microbe-disease associations cannot only reveal the pathogenesis of diseases, but also promote the diagnosis and prognosis of diseases. Because traditional medical experiments are time-consuming and expensive, many computational methods have been proposed in recent years to identify potential microbe-disease associations. In this study, we propose a method based on heterogeneous network and metapath aggregated graph neural network (MAGNN) to predict microbe-disease associations, called MATHNMDA. First, we introduce microbe-drug interactions, drug-disease associations, and microbe-disease associations to construct a microbe-drug-disease heterogeneous network. Then we take the heterogeneous network as input to MAGNN. Second, for each layer of MAGNN, we carry out intra-metapath aggregation with a multi-head attention mechanism to learn the structural and semantic information embedded in the target node context, the metapath-based neighbor nodes, and the context between them, by encoding the metapath instances under the metapath definition mode. We then use inter-metapath aggregation with an attention mechanism to combine the semantic information of all different metapaths. Third, we can get the final embedding of microbe nodes and disease nodes based on the output of the last layer in the MAGNN. Finally, we predict potential microbe-disease associations by reconstructing the microbe-disease association matrix. In addition, we evaluated the performance of MATHNMDA by comparing it with that of its variants, some state-of-the-art methods, and different datasets. The results suggest that MATHNMDA is an effective prediction method. The case studies on asthma, inflammatory bowel disease (IBD), and coronavirus disease 2019 (COVID-19) further validate the effectiveness of MATHNMDA.

## Introduction

The microorganisms related to the human body mainly include eukaryotes, archaea, bacteria, fungi, and viruses [Human Microbiome Project (HMP), 2012]. These microorganisms form different microbial communities and parasitize in different parts of the human body, such as the skin, mouth, genitalia, intestinal tract, and other parts. Studies have shown that the number of microbes in the adult intestine is equivalent to 10 times that of human cells (Sender et al., 2016), which indicates that the microbial community in the human body is relatively large. Microbes are generally beneficial to the human body. For example, by fermenting food ingredients that cannot be digested by the host, gut microbes can promote nutrient and energy absorption (Gill et al., 2006; Marco et al., 2017). The *Bifidobacteria* in the human intestine can produce lactic acid and acetic acid after fermentation, which can promote the absorption of iron and vitamin D. Therefore, a set of balanced microbes can keep the human body away from physiological disorders, but the imbalance or decline of the microbial community can harm the human host and cause diseases. For example, a study has found that compared to normal children, children with asthma would have a smaller number of *Faecalibacterium*, *Lachnospira*, *Veillonella*, and *Rothia* (Arrieta et al., 2015). Another study found that the relative abundance of *Enterococcus*, *Escherichia*/*Shigella*, *Klebsiella*, *Streptococcus*, and *Peptostreptococcus* in the intestinal flora of patients with colorectal cancer was increased (Wang et al., 2012). These studies have shown that identifying the relationship between microbes and diseases can help us understand the pathogenesis of the disease, so as to carry out more targeted treatment. Therefore, determining the relationship between microbes and diseases has become a key research topic in the current bioinformatics field.

Verifying the relationship between microbes and diseases through biological experiments is a time-consuming and expensive task. Therefore, many computational models have been proposed to predict the association between microbes and diseases. Wang et al. (2021) wrote a review on circular RNAs and complex diseases, which classified the prediction models of circRNA-disease associations. Inspired by this study, we can divide these computational models into four types according to the differences in the microbe-disease association prediction strategies based on heterogeneous networks: path-based methods, random walk methods, bipartite local models, and matrix decomposition methods (Wen et al., 2021). Path-based methods are widely used in association prediction (Zhang et al., 2021; Liu et al., 2022a). They make predictions by calculating path-based scores between microbe nodes and disease nodes. For example, Chen X. et al. (2016) proposed the first model KATZHMDA to predict microbe-disease associations, which calculated the predicted probability score according to the walking step length and walking times between the two nodes in the microbe-disease network. Huang et al. (2017) proposed a computational model PBHMDA based on the depth-first search to predict potential microbes associated with diseases. Fan et al. (2018) developed a new model MDPH_HMDA to predict microbe-disease associations by integrating multi-source data and path-based HeteSim score. The random walk has aroused extensive interest in the field of microbe-disease prediction. For instance, Yan et al. (2019) proposed a prediction model BRWMDA based on similarity and bi-random walk to predict potential microbe and disease associations. Luo and Long (2018) proposed a computational model NTSHMDA based on random walk and network topology similarity to predict the associations between microbes and diseases. Wu et al. (2018) developed a method named PRWHMDA, which attempted to infer potential microbe-disease pairs by random walk on the heterogeneous network with Particle Swarm Optimization (PSO). Bipartite local models are also common methods, which work independently on the basis of both sides of a microbe-disease pair and can be combined to yield a definitive prediction result. For example, Zou et al. (2018) proposed a method called NCPHMDA that utilized the network consistency projection to predict microbe-disease associations. Wang et al. (2017) constructed a semi-supervised computational model LRLSHMDA based on a Laplacian regularization least squares classifier to predict the associations between microbes and diseases. In addition, some prediction models for microbe-disease associations were developed based on matrix factorization techniques. For instance, He et al. (2018) presented a method called GRNMFHMDA, which incorporated weighted K-nearest known neighbors to predict microbe-disease associations. Shen et al. (2017) developed a computational model of CMFHMDA, which used collaborative matrix factorization to reconstruct the association matrices between diseases and microbes. Wang Y. et al. (2022) proposed a method HNGFL based on heterogeneous network and global graph feature learning to predict microbe-disease association. In addition to these computational models, several review articles on microbe-disease associations have been published. For example, Pan et al. (2022) developed a comprehensive approach to predict associations between genomics, proteinomics, transcriptomics, microbiome, metabolomics, pathomics, radiomics, drug, symptoms, environment factors, and disease networks. Wang L. et al. (2022) provided a comprehensive review on predicting pairwise relationships between human microbes, drugs, and diseases, from biological data to computational models. Wen et al. (2021) provided a survey on predicting microbe-disease associations based on biological data and computational methods.

Although the above-mentioned methods have achieved relatively stable prediction performance in the association prediction task of microbes and diseases, there are still some limitations and deficiencies. First, the vast majority of methods make predictions based on small-scale datasets, which makes them unable to obtain accurate predictions when it comes to new diseases (or new microbes) due to a lack of training data. Second, microbe imbalance (or the occurrence of disease) is not influenced by a single factor. Some studies have shown that microbes participate in drug absorption and metabolism, thereby regulating drug efficacy and drug toxicity for disease (Zimmermann et al., 2021). However, the above-mentioned methods are only based on microbes and diseases, which makes these models unable to obtain accurate prediction results due to the lack of more semantic information about microbes and diseases in the prediction process.

Therefore, with the discovery of multivariate biological data, the heterogeneous graph embedding method is increasingly applied to relational prediction. It can learn semantic and structural information between nodes to compensate for the poor prediction performance due to the small amount of known associated data. For example, Lei and Wang (2020) proposed a method based on Node2vec and a heterogeneous network scoring mechanism, called LGRSH, to predict the association between microbes and diseases. Liu et al. (2022b) proposed a method to identify miRNA-disease associations via deep forest ensemble learning based on autoencoder. Yang et al. (2022) proposed a DeepWalk-based method to predict lncRNA-miRNA associations via a lncRNA-miRNA-disease-protein-drug graph. Zhu et al. (2018) proposed a method using Metapath2vec to predict drug-gene interactions. Lei et al. (2021) developed a method, called CDWBMS, to predict circRNA-disease associations based on an improved weighted biased meta-structure. Zhang et al. (2020) adopted metapath2vec++ and matrix factorization to predict circRNA-disease associations. All the heterogeneous graph embedding methods have some limitations when applied to association prediction, such as ignoring the information of multiple nodes, discarding all intermediate nodes on the metapath, or only using a single metapath. This will affect the predictive performance of the model.

To deal with the above-mentioned issues, we developed a novel method based on a metapath aggregated graph neural network (MAGNN) and tripartite heterogeneous network for microbe-disease association prediction named MATHNMDA. In particular, we integrate information from different sources, such as microbe-disease associations, microbe-drug interactions, and disease-drug interactions, to construct a tripartite heterogeneous network of microbe-drug-disease. Further, we feed the heterogeneous network to MAGNN. For each layer of MAGNN, we first use intra-metapath aggregation with a multi-head attention mechanism to extract the structural and semantic information of the metapath instance. After that, we further apply inter-metapath aggregation with an attention mechanism to fuse latent vectors of multiple metapaths. Finally, we take the output of the MAGNN as the final embedding features of the microbe node and disease node, and make predictions. In order to verify the predictive performance of MATHNMDA, we carried out cross-validation experiments, and the results indicate that MATHNMDA can effectively identify potential disease-related microbes.

Overall, our main contributions are as follows:

(1) We expand known microbe-disease association data by integrating multiple databases, and construct a tripartite heterogeneous network by introducing drug-disease associations and microbe-drug associations. We further apply MAGNN to predict microbe-disease associations.
(2) We use intra-metapath aggregation with the multi-head attention mechanism to learn the topological information and semantic information embedded in the internal nodes of metapath, so that the embedding learned by the target node is more comprehensive.
(3) We use inter-metapath aggregation with an attention mechanism to aggregate the embeddings of different metapaths for target nodes (microbe nodes or disease nodes).
(4) We conduct a case study of coronavirus disease 2019 (COVID-19) to verify the effectiveness of the MATHNMDA model.

## Materials and Methods

### Dataset

**FIGURE 1:**
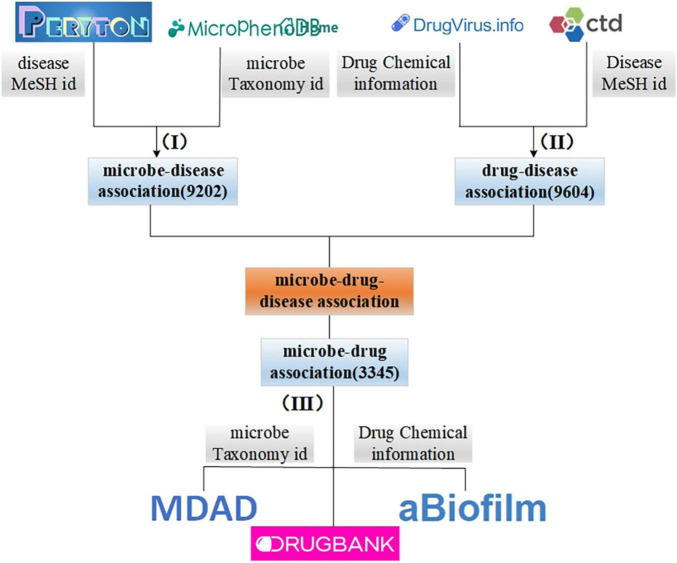
The processing and integration process of microbe-drug-disease association data.

In this study, we integrate the information obtained from different sources. First, we collect microbe and disease association data from Peryton (Skoufos et al., 2020) and MicroPhenoDB (Yao et al., 2021). Among them, Peryton includes more than 7,900 relationships between 43 diseases and 1,396 microbes. The data in MicroPhenoDB are collected from some public datasets, such as Human Microbe-Disease Association Database (HMDAD; Kong et al., 2017), Disbiome (Yorick et al., 2018), Virulence Factor Database (VFDB; Chen L. et al., 2016), etc. MicroPhenoDB has 5,565 relationships between 515 diseases and 1,717 microbes. After eliminating redundancy for the same diseases and microbes, we obtain a total of 9,202 associations between 538 diseases and 2,491 microbes. Furthermore, we collect data about microbes and their related drugs from Microbe-Drug Association Database (MDAD; Sun et al., 2018), drugVirus (Andersen et al., 2020), and aBiofilm (Akanksha et al., 2017), and remove redundant records to obtain a total of 132 microbes and 1,933 drugs and 3,345 microbe-drug associations. Then, we download disease-drug interaction data from drugBank (Wishart et al., 2017) and Comparative Toxicogenomics Database (CTD; Davis et al., 2012) databases, and we obtain 9,604 interactions between 127 diseases and 247 drugs after de-redundancy. Figure 1 illustrates the integration process of microbe-drug data, drug-disease data, and microbe-disease data. It is worth noting that in this study, we unified the disease, microbe, and drug according to the MESH id of the disease, the taxonomy id of the microbe and the chemical information of the drug, disease-related drugs, are included in drugs related to microbes.

### Construction of Microbe-Drug-Disease Tripartite Heterogeneous Network

**FIGURE 2:**
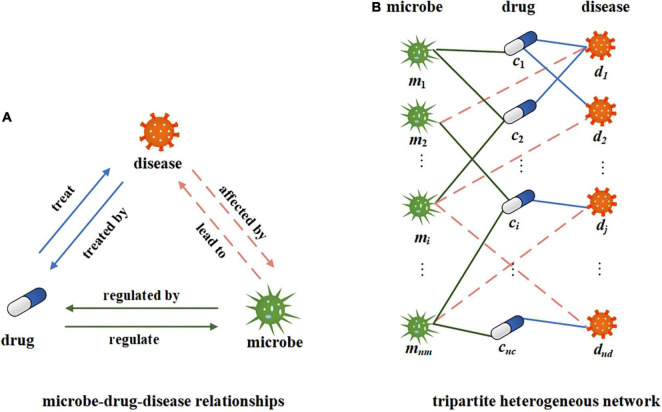
Illustration of microbe-drug-disease relationship and heterogeneous networks. **(A)** The illustration of microbe-drug-diease relationships. **(B)** A tripartite network corresponding to the microbe-drug-disease relationships.

In this study, we use microbe-disease, microbe-drug, and disease-drug associations to build a tripartite network. The relationship between microbes, drugs, and diseases is shown in Figure 2A. A certain microbial imbalance can lead to certain diseases. and the pathogenesis of a certain disease will be affected by certain microbial communities. Some drugs can treat some diseases, and certain diseases can be treated with certain drugs. Microbes can regulate the activity and toxicity of drugs (Zimmermann M. et al., 2019). Drugs in turn can change the diversity and function of microbial communities. Suppose *M*, *C*, and *D*, respectively, represent all the sets of microbes, drugs, and diseases in the network, *m*_*i*_∈*M* represents a microbe, *i* = 1, 2, 3…, *n*_*m*_; *c*_*j*_∈*C* represents a drug, *j* = 1, 2, 3…, *n*_*c*_; and *d*_*k*_∈*D* represents a disease, *k* = 1, 2, 3…, *n*_*d*_. Construct a tripartite heterogeneous network based on the relationship among microbes, drugs, and diseases. Here, we can simplify it to an undirected and unweighted network to represent the existence of associations, as shown in Figure 2B. We further construct the microbe-disease adjacency matrix $B\in R^{n_{m}\times n_{d}}$, where *n*_*m*_ represents the number of microbes and *n*_*d*_ represents the number of diseases. If there is a known association between a microbe node *i* and a disease node *j*, the value of *B*(*i*,*j*) is 1, otherwise, it is 0.

### MATHNMDA

**FIGURE 3:**
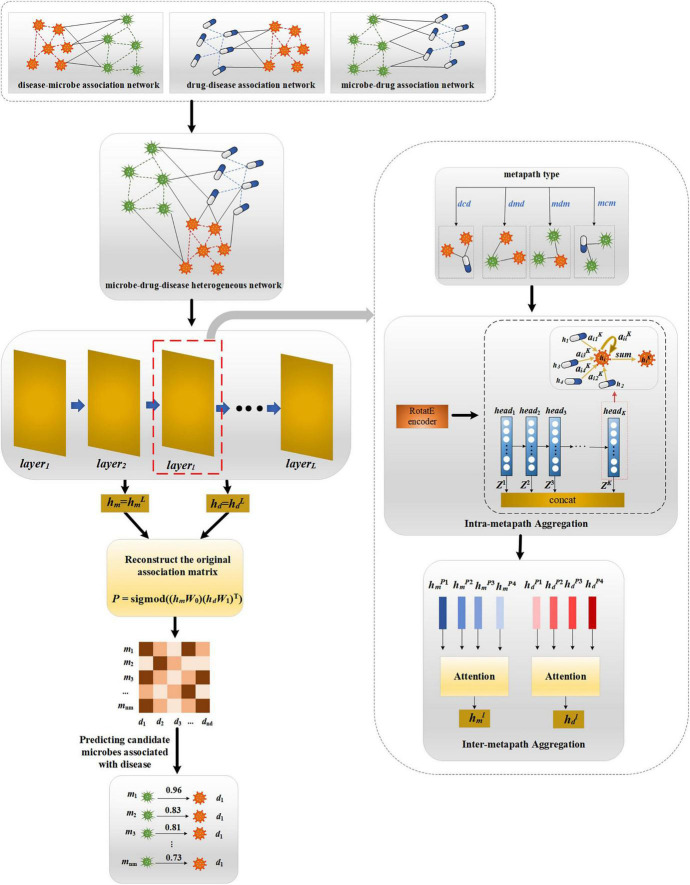
The framework of MATHNMDA. *mcm*, *mdm*, *dmd*, and *dcd* are four metapaths, where *m* represents microbe, *c* represents drug, and *d* represents disease.

Our proposed MATHNMDA model consists of three main steps, as shown in Figure 3. The model takes heterogeneous microbe-drug-disease interaction network and MAGNN to predict microbe-disease associations. First, we take heterogeneous network as input of the MAGNN. Second, for each layer of the MAGNN, we use intra-metapath aggregation to learn the structural and semantic information embedded in the target node, metapath-based neighbor nodes, and the context between them. Third, we apply inter-metapath aggregation to combine the semantic information of all different metapaths. Finally, we take the output of the MAGNN as vector representations of microbe nodes and disease nodes, which can be used to predict potential microbe-disease associations.

### Intra-Metapath Aggregation

In this study, we predict novel microbe-disease associations on the heterogeneous microbe-drug-disease interaction networks based on MAGNN. Given a heterogeneous network *G* = (*V*, *E*), where *V* and *E* represent sets of nodes and edges, respectively, and the mapping functions of nodes and edges are δ: *V*→*A* and ψ: *E*→*R*, *A* represents node types, *R* denotes edge types, and |*A*| +|*R*| >2. Given a metapath *M* on the heterogeneous network *G*, we can define it as a path of the form *A*_1_→*A*_2_→…→*A*_*n*–1_→*A*_*n*_, which can be abbreviated as *A*_1_*A*_2_…*A*_*n*–1_*A*_*n*_. The relationship between node types *A*_1_ and *A*_*n*_ is *R* = *R*_1_◦*R*_2_◦…◦*R*_*n*–1_, where ° represents the composite operation. That is to say, the relationship *R* is obtained by compositing the *n*–1 relationships of these *R*_1_, *R*_2_, …, *R*_*n*–1_. Therefore, a metapath can capture specific semantic information in the graph, and different metapaths represent different semantic information. For example, for the metapath microbe-drug-disease (abbreviated as *m*-*c*-*d*), drug *c* can act on microbe *m*, and drug *c* can be used to treat disease *d*, so microbe *m* may be associated with disease *d*. The key idea of intra-metapath aggregation is to learn structural and semantic information embedded in target nodes, metapath-based neighbors, and the context between them by encoding metapath instances under a certain metapath. Next, we introduce the process of intra-metapath aggregation in detail.

Given a metapath *M*, we define a sequence of nodes in *G* that follow the pattern of *M* as a metapath instance, defined as *M*(*i,j*), which is represented as a metapath instance connecting the target node *i* and its neighbor node *j* based on the metapath. Here, $j\in N_{i}^{M}$, $N_{i}^{M}$ represents the set of nodes connected to node *i* through the metapath instance *M*(*i,j*). It is worth noting that if the metapath instance *M*(*i,j*) is symmetric, $j\in N_{i}^{M}$ also includes node *i* itself. Then we define the intermediate node set of *M*(*i,j*) as *TH*(*i,j*), *TH*(*i*, *j*) = *M*(*i*, *j*)/{*j*, *i*}, where {*j*, *i*} represents the set with elements *i*, *j*.

As mentioned before, intra-metapath aggregation learns structural and semantic information of target nodes by encoding metapath instances. Sun et al. (2019) proposed a method for knowledge graph embedding based on relational rotation in complex space, called RotatE. RotatE can model all relational patterns, so we use RotatE as the metapath instance encoder in this study. Given a metapath instance *M*(*i,j*) = (*i*, *th*_2_…, *th*_*n*–1_, *j*), for convenience, let set *i* = *th*_1_ and *j* = *th*_*n*_. *R*_*i*_ represents the relationship between node *th*_*i*_ and node *th*_*i*+1_, and the relationship vector is *r*_*i*_. Therefore, for the metapath instance *M*(*i,j*), RotatE can be defined as follows:


(1) $$\begin{array}{c} \Theta_{1}={\tilde{h}}_{{\mathrm{th}}_{1}}={\tilde{h}}_{i}\\ \Theta_{i}={\tilde{h}}_{{\mathrm{th}}_{i}}+\theta_{i-1}⊙r_{i}\\ h_{M\,\left(i,j\right)}=\frac{\theta_{n}}{n} \end{array}$$

where ${\tilde{h}}_{t\,h_{i}}$ and *r*_*i*_ are vectors of complex space, ⊙ represents hadamard product, $hM(i,j)\in R^{d^{'}}$, and $d^{'}$ is the dimension of *h*_*M*(_*_*i*_*,*_*j*_*_)_. In which case, we get vector representation of the metapath instance *M*(*i,j*). It is important to note that there may be multiple instances of the metapath *M* connecting nodes *i* and *j*, but we use *M*(*i,j*) to represent a single instance here.

Graph attention network (GAT) is an effective graph representation learning tool, which represents the importance of neighbor nodes to the target node by assigning different weights to different neighbor nodes (Bian et al., 2021). Here, for target node *i* and metapath *M* related to *i*, we first use GAT to assign weights (attention coefficients) to metapath instances in *M*, thereby learning the importance of different metapath instances to target nodes. Then the features of different metapath instances are aggregated according to the obtained attention coefficients, which are represented as the feature vector of the target node *i*. Given a metapath instance *M*(*i*,*j*), its attention coefficient can be defined as:


(2) $$e_{\mathrm{ij}}^{M}=\mathrm{LeakyReLU}\,\left(\delta_{M}^{T}\,\left[{\tilde{h}}_{i}∥h_{M\,\left(i,j\right)}\right]\right)$$

where $\delta_{M}^{T}$ is the attention parameter of the metapath *M*, and ∥ represents connection operation. To make the attention coefficients of different metapath instances comparable, we use the softmax function to normalize $e_{i\,j}^{M}$:


(3) $$\alpha_{\mathrm{ij}}^{M}=\mathrm{softmax}\,\left(e_{\mathrm{ij}}^{M}\right)=\frac{\exp\,\left(e_{\mathrm{ij}}^{M}\right)}{\sum_{k\in {\text{N}}_{\text{i}}^{\text{M}}}\text{exp}\,\left({\text{e}}_{\text{ij}}^{\text{M}}\right)}$$

Then, we aggregate the feature vectors of all metapath instances according to the activation function σ(⋅) to obtain the vector representation of node *i* based on the metapath *M*:


(4) $$\alpha_{\mathrm{ij}}^{M}=\sigma \,\left(\sum_{j\in {\text{N}}_{\text{i}}^{\text{M}}}\alpha_{\text{ij}}^{\text{M}}\cdot {\text{h}}_{\text{M}\,\left(\text{i},\text{j}\right)}\right)$$

In this study, we further introduce a multi-head attention mechanism to stabilize the learning process of attention coefficients and reduce the influence of a single attention. Specifically, we independently repeat the attention mechanism *K* times and concatenate vector representation learned by each attention head. Therefore, the vector representation of node *i* can be further rewritten as follows:


(5) $$h_{i}^{M}=\begin{array}{c} K\\ ∥\\ k=1 \end{array}\,\sigma \,\left(\sum_{j\in {\text{N}}_{\text{i}}^{\text{M}}}\alpha_{\text{ij}}^{\text{M}}\cdot {\text{h}}_{\text{M}\,\left(\text{i},\text{j}\right)}\right)\,$$

Since the metapath is undirected, each node in the metapath can be either a start node or an end node. Therefore, for the metapath set starting or ending with node type *a* ∈ *A*, it is denoted as *M*_*a*_ = {*M*_1_, *M*_2_,⋯, *M*_*S*_}, and *S* represents the number of metapaths. Intra-metapath aggregation obtains *M* metapath-specific vector representations of the target node *i* ∈ *V*_*a*_, defined as $\left\{h_{i}^{M_{1}},h_{i}^{M_{2}},\cdots ,h_{i}^{M_{S}}\right\}$.

### Inter-Metapath Aggregation

After the intra-metapath aggregation of metapaths, we obtain the vector representation of a single metapath *M* for target node *i*. Then, we need to aggregate the semantic information and structural information of node *i* based on all metapaths of *M*_*a*_, where *S* represents the number of metapaths. The node embedding set corresponding to these *S* metapaths is $\left\{h_{i}^{M_{1}},h_{i}^{M_{2}},\cdots ,h_{i}^{M_{S}}\right\}$. A simple aggregation method between metapaths is to take the average of these node embeddings. However, because the importance of metapaths to node *i* in a heterogeneous network is different, we allocate weight for each metapath pattern through the attention mechanism, and then perform aggregation.

Specifically, given a metapath *M*_*p*_, *M*_*p*_ ∈ *V*_*a*_, we first transform these metapath-specific node vectors for all nodes *i* ∈ *V*_*a*_ with the tanh function, and then take average value as feature of *M*_*p*_:


(6) $$S_{M_{p}}=\frac{1}{\left|V_{a}\right|}\,\sum_{i\in V_{a}}\tanh\,\left(W_{a}\cdot h_{i}^{M_{p}}+b_{a}\right)$$

where *W*_*a*_ is the weight matrix of nonlinear transformation specific to node type *a*, and *B*_*a*_ is the corresponding bias, both of which are learnable parameters. *V*_*a*_ indicates all nodes of type *a* in the network.

Then we use the attention mechanism to calculate the importance of each metapath pattern for the target node *i*, and normalize the obtained attention coefficients by the softmax function. Then we fuse the corresponding vector representations of these metapaths to get the output of the target node *i*, as shown in Equation 7:


(7) $$\begin{array}{c} e_{M_{p}}=c_{a}^{T}\cdot S_{M_{p}}\\ \beta_{M_{p}}=\mathrm{softmax}\,\left(e_{M_{p}}\right)=\frac{\exp\,\left(e_{M_{p}}\right)}{\sum_{M_{p}\in {\text{M}}_{\text{a}}}\text{exp}\,\left({\text{e}}_{{\text{M}}_{\text{p}}}\right)}\,\\ {\text{h}}_{\text{i}}^{{\text{M}}_{\text{a}}}=\sum_{M_{p}\in M_{a}}\beta_{{\text{M}}_{\text{p}}}\cdot {\text{h}}_{\text{i}}^{{\text{M}}_{\text{p}}}\, \end{array}$$

where $c_{a}^{T}$ denotes the attention parameter, ${\text{β}}_{M_{P}}$ denotes the normalized attention score, and *M*_*P*_ denotes the *P*th metapath in *M*_*a*_. $h_{i}^{M_{a}}$ represents the embedding vector of node *i* based on aggregation between metapaths.

### MAGNN

The goal of a graph neural network (GNN) is to learn the low-dimensional vector representation of each node, which can be used for many downstream tasks, such as node clustering, node classification, and link prediction. Thus, we further apply an *L*-layer GNN to learn the low-dimensional representation vectors of microbe nodes and disease nodes. At each layer of the GNN, we use intra-metapath aggregation and inter-metapath aggregation to obtain vector representations of node-based metapath. In this way, we can define the low-dimensional representation for node *i* at the *l*th layer:


(8) $$h_{i}^{l}=\sigma \,\left(W_{o}^{l}\cdot {\left[h_{i}^{M_{a}}\right]}^{l}\right)$$

where σ(⋅) is an activation function and $W_{o}^{l}$ represents the weight vector at the *l*th layer. $\,h_{i}^{l}$ represents the vector representation for node *i* at the *l*th layer, which is also the input of the (*l*+1)th layer. We define $h_{i}^{0}=W_{a}\cdot X_{i}^{a}$, where *W*_*a*_ represents the linear transformation matrix of node type *a* and *X*_*i*_ is the original feature vector for node of type *a*. Here, we use one-hot encoding to initialize each type of node in the heterogeneous network.

Finally, we use vector representation of node *i* at the *L*th layer to serve as the final embedding for nodes *i*:


(9) $$h_{i}=h_{i}^{L}$$

where $h_{i}^{L}$ represents vector representation of node *i* at the *L*th layer.

### Reconstruction of Microbe-Disease Association

After we get the final embeddings of all microbe nodes and disease nodes, we can predict new microbe-disease associations by reconstructing microbe-disease associations. Here we perform a simple inner product operation on the microbe and disease embeddings. In this case, each microbe-disease pair will receive a new score. Specifically, given a microbe node *m* and a disease node *d*, the predicted score *C*_*md*_ between them can be calculated as:


(10) $$C_{\mathrm{md}}=\mathrm{sigmoid}\,\left(h_{m}^{T}\cdot h_{d}\right)$$

where *h*_*m*_ and *h*_*d*_ represent the final embeddings of microbes and diseases, respectively.

### Optimization

Since our task is to predict microbe-disease associations, this is equivalent to a binary classification problem. So, here we use the cross-entropy function as the loss function and optimize through negative sampling:


(11) $$L=-\sum_{\left(m,d\right)\in \mu }\log\,\left(C_{\mathrm{md}}\right)-\sum_{\left(m,d\right)\in \mu^{-}}\log\,\left(-C_{\mathrm{md}}\right)$$

where μ represents the set of positive samples, and μ^–^ represents the set of negative samples obtained by negative sampling.

## Results

In this section, we evaluate the performance of MATMNMDA through some experiments and analysis of the results. At the same time, we also analyze and adjust some parameters of the model in order to make better predictions.

### Evaluation Metrics

In this study, we mainly use two metrics to evaluate the performance of the model, area under the receiver operating characteristic curve (AUC) and area under the precision–recall curve (AUPR), which are widely used in association prediction tasks.

*AUC*: This corresponds to the area of a planar graph bounded by the receiver operating characteristic (ROC) curve and horizontal axis, which can estimate the performance of binary classification models. The value of AUC is between 0 and 1. When it is closer to 1, the model performs better. In practical application, the advantages and disadvantages of different models can be compared by comparing the AUC values of different classification models.

*AUPR*: The precision–recall (PR) curve is also used to evaluate the classification ability of the model. In particular, the PR curve can collect more information when dealing with some imbalanced datasets. The area enclosed by the PR curve and the abscissa axis is called AUPR.

### Baselines

In order to test the effectiveness of the MATMNMDA model, we compare it with six state-of-the-art methods based on the data processed in this study. Here, we calculate the AUC and AUPR values of these methods under the same conditions and analyze the results. The six baselines are as follows:

BRWMDA (Yan et al., 2019): It is a similarity-based and modified bi-random walk to predict associations between microbes and diseases.

KATZHMDA (Chen X. et al., 2016): It is a method to predict microbe-disease associations based on the katz metric.

LRLSHMDA (Wang et al., 2017): It is a semi-supervised model to predict microbe-disease associations by introducing a Gaussian kernel and Laplacian regularization.

NCPHMDA (Zou et al., 2018): It uses network consistent projections to predict microbe-disease associations.

NTSHMDA (Luo and Long, 2018): Predicting microbe-disease associations using heterogeneous network topological similarity and random walks.

CRPGCN (Ma et al., 2021): It is a method based on graph convolutional network (GCN) and random walk with restart (RWR), which was proposed for the cirRNA-disease association prediction task. Here, we use it as a baseline method for the prediction of microbe-disease association.

We compare MATMNMDA with these six baseline methods under the same conditions. For the CRPGCN method, the similarity of microbes and diseases is calculated in the same way as BRWMDA. For the MATMNMDA model, we first perform negative sampling on the microbe-drug-disease heterogeneous network. The positive and negative sample ratios of the training set, validation set, and test set are 1:1, and the proportion of the training set, validation set, and test set is 8:1:1, respectively. We randomly initialize vector representations of microbe nodes, drug nodes, and disease nodes. The Adam optimizer is used to optimize the model. The dropout and early stopping mechanisms are used to prevent overfitting. Here, according to the extensive literature (Phaisangittisagul, 2016), we set the value of dropout to 0.5. We train the model 100 times.

### Parameter Analysis

In this section, we analyze the sensitivity of parameters. As we all know, important parameters will affect the performance of the model, so it is very necessary to conduct parameter analysis for the model. Some important parameters involved in the MATMNMDA model include the dimension of hidden layer, number of heads in the multi-head attention mechanism, dimension of attention vector, and number of neighbors sampled by the nodes in the experiment. We analyze these four parameters in turn and evaluate their impact on model performance.

**FIGURE 4:**
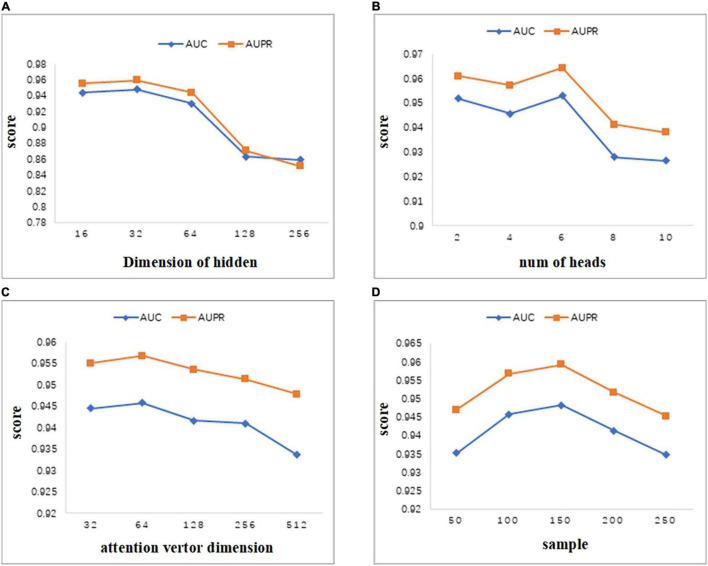
Parameter analysis. **(A)** Comparison of AUC and AUPR for different hidden layer dimensions. **(B)** Comparison of AUC and AUPR of attention heads for different multi-attention mechanisms. **(C)** Comparison of AUC and AUPR for different attention vector dimensions. **(D)** Comparison of AUC and AUPR for different numbers of neighbors sampled by nodes.

As can be seen from Figure 4A, we set the dimension of the hidden layer to 16, 32, 64, 128, 256. As the dimension of the hidden layer increases, the performance of the model first increases. When the dimension reaches 32, both AUC and AUPR reach the maximum value. As the dimension continues to increase, the performance of the model begins to decrease gradually. Therefore, in this study, we set the embedding dimension of the hidden layer as 32. When the dimension changes between 16 and 256, the values of AUC and AUPR vary greatly. Thus, the MATMNMDA model is sensitive to the dimension of the hidden layer.

MATMNMDA model adopts a multi-attention mechanism to stabilize the process of attention coefficient learning. Figure 4B shows the influence of the number of attention heads in the multi-attention mechanism on model performance. We change the number of attention heads from 2 to 10 by step 2. It can be seen that when the number of attention heads is set to 6, the model has the best performance. Figure 4C shows the influence of the dimension of the attention vector. The dimension of the attention vector changes between 32 and 512. It can be observed that the vector dimension is too small or too large, which is not good for the performance of the model. Specifically, if the dimension of the attention vector is too large, it may lead to overfitting, which will degrade the performance of the model. When the dimension is set to 64, we can obtain better prediction ability.

In the MATMNMDA model, intra-metapath aggregation involves aggregating features of neighbor nodes to represent the representation of the current target node. Therefore, we analyze the number of neighbor nodes. In Figure 4D, the number of neighbor nodes is selected from {50,100,150,200,250}. It can be seen that when the number of neighbor nodes is too small or too large, the performance of the model is not very good. Specifically, if the number of neighbor nodes is too small, the structural information and semantic information of the target node may not be so comprehensive, while too large may cause noise. Therefore, we set the number of neighbor nodes to 150.

### Ablation Study

As mentioned in the Introduction section, the previous heterogeneous network embedding methods have the following problems: (1) They only consider the neighbors based on the metapath, and do not consider the intermediate nodes inside the metapath. (2) In the metapath-based embedding, only the single best metapath is considered, and our model is proposed based on these problems. Therefore, in order to verify the effectiveness of each module of our model, we further conduct experiments on different variants of the MATMNMDA model. Taking MATMNMDA as a reference model, here we tested three variants of it.

MATMNMDA_nb: It only considers metapath-based neighbor nodes and does not consider intermediate nodes.

MATMNMDA_sm: It only considers the single best metapath.

MATMNMDA_avg: It replaces the RotatE with a mean encoder.

**FIGURE 5:**
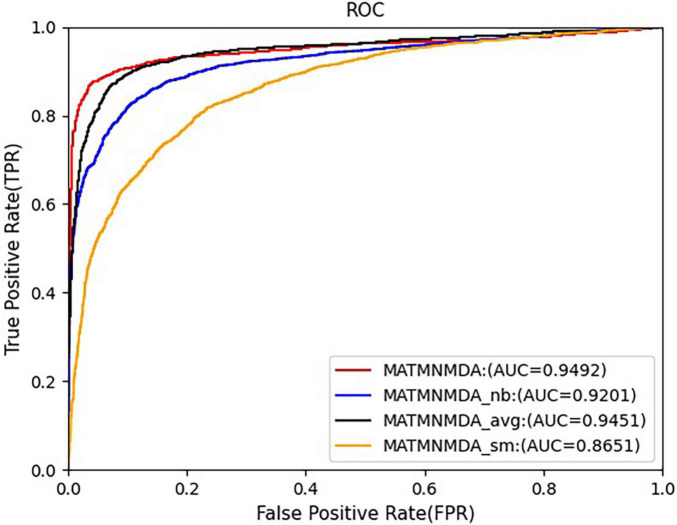
Comparison of AUC for MATMNMDA and its variants.

**FIGURE 6:**
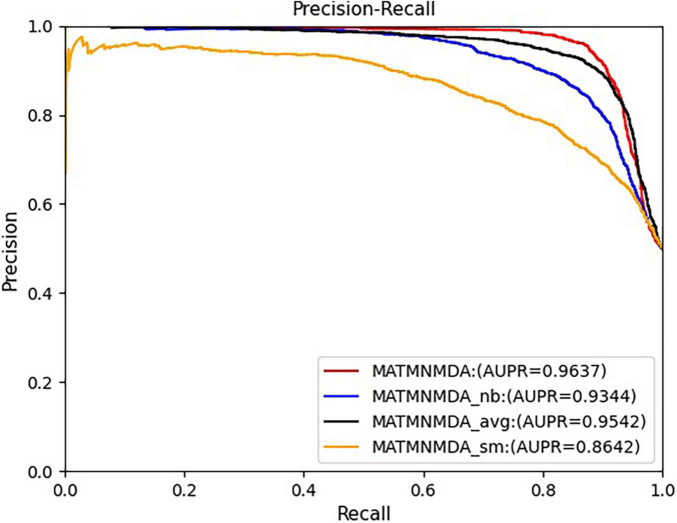
Comparison of AUPR for MATMNMDA and its variants.

Figures 5, 6 show the comparison results of the MATMNMDA model and its variants. We can see that the MATMNMDA model has the highest AUC and AUPR. Followed by MATMNMDA_avg, MATMNMDA_sm has the worst performance. Comparing MATMNMDA and MATMNMDA_avg, we find that the MATMNMDA model performs better, which is because the mean encoder essentially treats metapath instances as a set and ignores the information embedded in the sequential structure of metapaths, while RotatE can be modeled according to the sequential structure of metapaths, thereby preserving the information embedded in the sequential structure of metapaths, so RotatE helps to improve the performance of the model by a small amount. Comparing MATMNMDA and MATMNMDA_nb, we can find that considering the intermediate nodes inside the metapath can help the model to obtain more structural information and thus improve the performance of the model. The results of MATMNMDA and MATMNMDA_sm show that the model performance can be significantly improved by combining multiple metapaths.

### Comparison With Baselines

**FIGURE 7:**
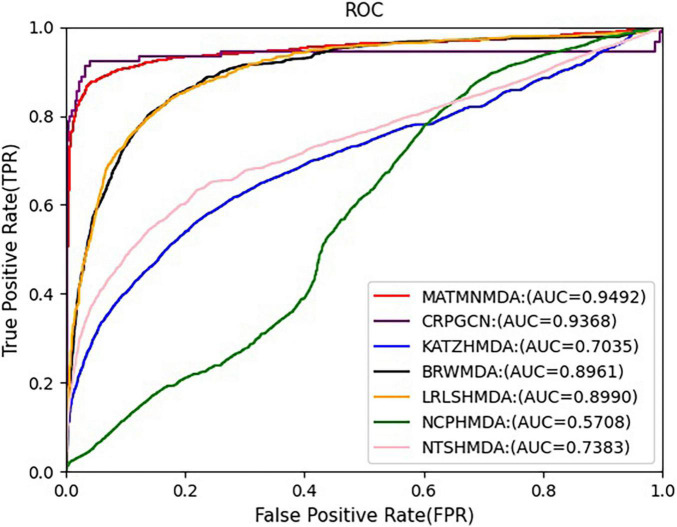
Comparison of AUC for MATMNMDA and baselines.

**FIGURE 8:**
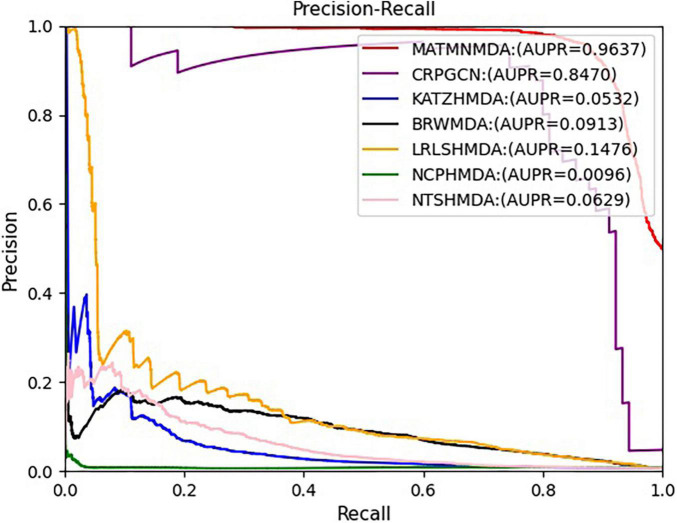
Comparison of AUPR for MATMNMDA and baselines.

We run these baseline methods with default parameters. Figures 7, 8 show the performance of different methods. Our model achieves the highest prediction results on these two evaluation metrics, and its AUC and AUPR reach 0.9492 and 0.9637, respectively, which are better than all baseline methods. The CRPGCN model occupies the second position. It applies the RWR algorithm, which allows each calculated node to better fuse information from neighboring nodes with higher weights, so that GCN can learn features faster and get higher prediction scores. Next is the LRLSHMDA model, because the topological structure in the microbe-disease association network helps the model to effectively use the hidden information of vertices and edges, which helps to train the optimal classifier, so that microbe-disease associations can be predicted more accurately. Next is the BRWMDA model, which also achieved good prediction results, because the BRWMDA model is based on similarity and bi-random walk, and it can model the topology information of the network well. However, NCPHMDA and NTSHMDA have poor prediction performance, because although we have obtained 9,202 known microbe-disease associations, they account for 0.7% of the whole microbe-disease association. The whole network is very sparse, and these two methods are based on network structure, so their performance is relatively poor.

### Comparison With Different Datasets

**FIGURE 9:**
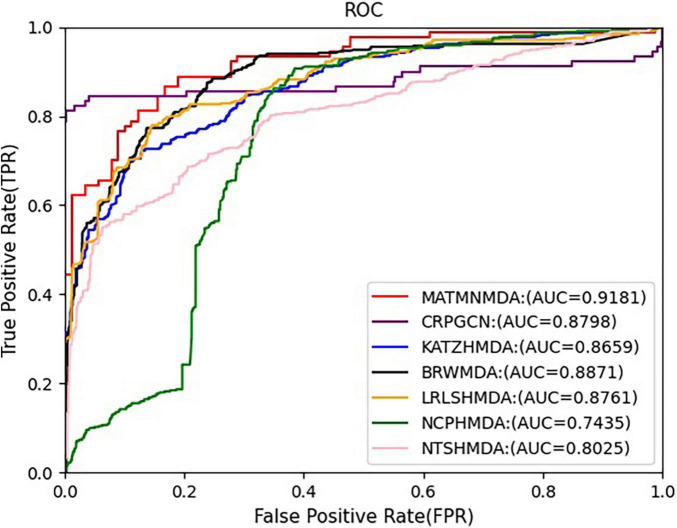
Comparison of AUC for MATMNMDA and baselines on HMDAD dataset.

**FIGURE 10:**
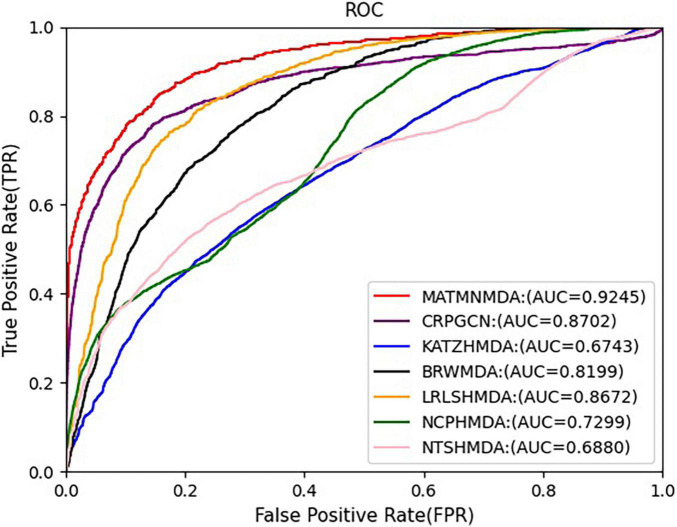
Comparison of AUC for MATMNMDA and baselines on Disbiome dataset.

In this study, we augment the known microbe-disease association data. In order to verify the validity of the MATHNMDA model in our dataset, we also compare MATHNMDA with baseline methods on HMDAD and Disbiome, which are commonly used in microbe-disease prediction. The results are shown in Figures 9, 10, and the comparison results of these methods on the three datasets are shown in Table 1. From Table 1, we can see that on each dataset, our model achieves the highest prediction value. It performs best on our dataset, so we can suggest that augmenting the known microbe-disease associations can help to improve the performance of the MATHNMDA. In addition, we can see that CRPGCN, LRLSHMDA, and BRWMDA methods perform well on these three datasets among the baseline methods. It also shows that these three methods are suitable for both large and small datasets, and the robustness of models is better. The remaining comparison methods are only suitable for small datasets.

**TABLE 1:** Performance comparison of MATMNMDA and baselines on different datasets.

DATASET	HMDAD		Disbiome		Our dataset	
METHOD	AUC	AUPR	AUC	AUPR	AUC	AUPR
CRPGCN	0.8798	0.4533	0.8702	0.4965	0.9368	0.8470
KATZHMDA	0.8815	0.4828	0.6743	0.0508	0.7035	0.0532
BRWMDA	0.8748	0.3966	0.8199	0.0705	0.8961	0.0913
LRLSHMDA	0.8766	0.4960	0.8672	0.1370	0.8990	0.1476
NCPHMDA	0.7524	0.0795	0.7299	0.1024	0.5708	0.0092
NTSHMDA	0.8276	0.2975	0.6880	0.0630	0.7383	0.0629
*MATMNMDA*	0.9181	0.9297	0.9245	0.9322	0.9492	0.9637

### Case Study

To further evaluate the predictive ability of the MATMNMDA model in identifying new microbe-disease associations, we conduct case studies on asthma, inflammatory bowel disease (IBD), and COVID-19. For each disease, microbes that have known associations with the disease are first removed. Then the predicted scores of candidate microbes are sorted in descending order according to the MATMNMDA model. Finally, we verify whether the top 10 microbes associated with the disease are confirmed by the relevant literature.

Asthma is a heterogeneous disease characterized by chronic airway inflammation (Lee and Kim, 2021). More than 300 million people worldwide suffer from asthma, and the incidence of asthma increased by 12.6% between 1990 and 2015 (Vasily, 2017). Therefore, it is necessary to study asthma deeply. With the development of 16rRNA sequencing technology, it has been found that there is an important relationship between asthma and microbe. In this study, when we employ the MATMNMDA model to predict potential microbe-disease associations, 7 of the top 10 candidate microbes are verified by relevant literature in PubMed (as shown in Table 2). For example, studies have shown that *Staphylococcus* (2nd) is linked to asthma attacks (Zhou et al., 2019), the relative abundance of *Bacteroidetes, Clostridium* (3rd), and *Enterobacteriaceae* were high, and the relative abundance of *Bifidobacterium* and *Lactobacteriaceae* were low, which is associated with allergies, eczema, or asthma (Zimmermann P. et al., 2019). An increased prevalence of *Staphylococcus aureus* (6th) colonization and sensitivity against its proteins are found in asthma (Tomassen et al., 2013). Bacterial dysbiosis and abundance within *Firmicutes* (4th) were significantly reduced in asthmatic children (Hufnagl et al., 2020). *Human parainfluenza virus* 1 (4th) was detected most frequently from patients with URI (3.74%, 47/1,257), followed by those with bronchitis (2.14%, 53/2,479), pneumonia (0.85%, 145/17,068), bronchiolitis (0.47%, 12/2,536), and asthma (0.43%, 2/462; Wang et al., 2015). *Herpesviruses* were the most abundant virus type in the asthma group (44.6 ± 4.6%), mainly *cytomegalovirus* (CMV; 9th) and EBV, which accounted for 24.5 ± 3.3 and 16.9 ± 3.5%, respectively (Choi et al., 2021). In healthy controls, the two viruses were 5.4 ± 2.5 and 7.1 ± 3.0%, respectively. Therefore, CMV and EBV are more abundant in patients with asthma exacerbations.

**TABLE 2:** Top 10 candidate microbes related to asthma.

Rank	Microbe	Evidence
1	Geotrichum sp.	PMID: 9376049
2	Staphylococcus	PMID: 24117882
3	Clostridiaceae	PMID: 30600099
4	Firmicutes	PMID: 32072252
5	Mitsuokella	Unconfirmed
6	Staphylococcus aureus	PMID: 30193937
7	Human parainfluenza virus 1	PMID: 26481737
8	Sphingobacteriia	Unconfirmed
9	Cytomegalovirus	PMID: 33757721
10	Aeromonas hydrophila	Unconfirmed

Inflammatory bowel disease (IBD) is an idiopathic intestinal inflammatory disease, mainly including ulcerative colitis (UC) and Crohn’s disease (CD), with clinical manifestations of abdominal pain, diarrhea, and bloody stools. It is difficult to completely cure the disease, which is easy to recur, and there is a potential risk of cancer. Therefore, we perform a case study of IBD to evaluate the predictive ability of the MATMNMDA model for novel microbe-disease associations. The results are shown in Table 3, and 7 of the top 10 candidate microbes are verified by relevant literature. For example, *Fusobacterium* (2nd), *Halomonas*, *Acinetobacter*, *Shewanella*, and *Streptococcus* were enriched in the CD microbiota (Weng et al., 2019). Increased abundance of *Salmonella* sp., *Campylobacter* sp., *Helicobacter* sp., *Escherichia coli*, *Alcaligenes* sp., and *Mycobacterium* sp. (4th) was observed in IBD patients (Olejniczak-Staruch et al., 2021). IBD patients exhibit a lower abundance of butyrate-producing bacteria (6th; Gasaly et al., 2021) and butyrate content. Although some findings related to IBD dysbiosis have varied among the studies due to differences in sample type, survey method, patient profile, and drug treatment, the most consistent observation across these studies is that bacterial diversity decreased in IBD patients. For viruses infecting human cells, *Anelloviridae* (5th) showed a higher prevalence in very early-onset IBD compared to healthy controls (Liang et al., 2020). The population of *Firmicutes* decreased (7th) and that of *Proteobacteria* increased (Matsuoka and Kanai, 2015). Researchers observed a bias in the fungal microbiota in IBD compared to the normal control group, with an increased *Basidiomycota*/*Ascomycota* ratio (8th), a decreased *Saccharomyces cerevisiae* ratio, and an increased *Candida albicans* ratio (Sokol et al., 2017). There are experiments to verify that the intensity of both CMV and *human herpesvirus* 6 (HHV-6; 9th) correlated with endoscopic disease severity in IBD (CMV, *p* = 0.010 and HHV-6, *p* = 0.048; Sipponen et al., 2011).

**TABLE 3:** Top 10 candidate microbes related to IBD.

Rank	Microbe	Evidence
1	Holophagae	Unconfirmed
2	Fusobacterium	PMID: 31240835
3	Sneathia sanguinegens	Unconfirmed
4	Mycobacterium sp.	PMID: 33924414
5	Anelloviridae	PMID: 32406906
6	Butyrate-producing bacterium	PMID: 33802759
7	Firmicutes	PMID: 25420450
8	ascomycota	PMID: 26843508
9	Human herpesvirus 6	PMID: 21879802
10	Nitrososphaeraceae	Unconfirmed

Coronavirus disease 2019 (COVID-19) is a disease caused by severe respiratory syndrome coronavirus 2 (SARS-CoV-2). It has been 3 years since its emergence and has become a pandemic threat to human health and the world economy. Although most cases of COVID-19 are mild or moderate, 3–4% of patients may be severe or critical, leading to hospitalization, respiratory failure, or death (Shen et al., 2020; Taleghani and Taghipour, 2021). Recent studies have found significant changes in the gut microbiome after infection with SARS-CoV-2. Therefore, this study conducts a case study on COVID-19 to evaluate the predictive ability of the model for COVID-19-related microbes, thereby helping researchers to conduct experimental verification purposefully, thus saving manpower and material resources. The results are presented in Tables 3, 4, of the top 10 candidate microbes were verified by relevant literature. For example, the analysis of fecal samples from COVID-19 patients found that the populations of *Bacteroides dorei*, *Bacteroides thetaiotaomicron* (5th), *Bacteroides massiliensis*, and *Bacteroides ovatus* were negatively associated with SARS-CoV-2 viral load in the samples (Zuo et al., 2020). Mycological analysis revealed that 77.8 and 30.6% of patients were infected with *Mucor* (8th) and *Aspergillus*, respectively (El-Kholy and El-Fattah, 2021). *Staphylococcus haemolyticus*, *Prevotella disiens* (9th), and 2 *Corynebacterium*_1 unclassified amplicon sequence variants were more abundant in people with low SARS-CoV-2 viral load during COVID-19 infection (Rosas-Salazar et al., 2021).

**TABLE 4:** Top 10 candidate microbes related to COVID-19.

Rank	Microbe	Evidence
1	Dyella	Unconfirmed
2	Acinetobacter calcoaceticus	Unconfirmed
3	Coriobacteriaceae bacterium	Unconfirmed
4	Bacteroides intestinalis	Unconfirmed
5	Bacteroides thetaiotaomicron	PMID: 32442562
6	Pisolithaceae	Unconfirmed
7	Pigmentiphaga	Unconfirmed
8	Mucor	PMID: 34009676
9	Prevotella disiens	PMID: 33577896
10	Blumeria graminis	Unconfirmed

## Conclusion

Increasing studies have shown that microbes play a key role in human health and disease. Microbe-disease associations cannot only reveal disease pathogenesis, but also promote the diagnosis and prognosis of diseases. Therefore, research on microbe-disease associations has attracted wide attention. In this study, we propose a novel computational model, called MATMNMDA, to predict potential microbe-disease associations. In order to capture more semantic and structural information between microbe nodes and disease nodes, we introduce drugs to construct a tripartite heterogeneous network and apply MAGNN to learn low-dimensional embedded representations of microbe nodes and disease nodes. For each layer of MAGNN, we use intra-metapath aggregation to get the representation of the target node in each metapath, which is the input of inter-metapath aggregation layer. Then we aggregate the embedding representations between different metapaths related to the target node. Therefore, we can learn the embedding representation for the target node (microbe node or disease node) of the layer. Finally, we obtain vector representations of microbes and diseases based on the output of the last layer in the MAGNN, which is used for the prediction task. We designed multiple experiments to verify the effectiveness of the MATMNMDA model. By analyzing the experimental results, we found that: (1) Compared to the variants of our model, our model obtains the best prediction performance, which also indicates that our method could be better applied to microbe-disease prediction. (2) Under the same conditions, compared to the state-of-the-art methods, our method also obtains the best AUC and AUPR, which indicates that the MATMNMDA model can better identify potential disease-related microbes. (3) Compared to the state-of-the-art methods on different datasets, MATMNMDA achieves the best prediction performance on our enlarged dataset. It demonstrates that more known microbe-disease associations can help MATHMDA improve predictive performance. (4) Case studies on asthma, IBD, and COVID-19 further verified the effectiveness of MATMNMDA.

In future work, we will add more relational data, such as drug-drug interactions, drug-protein interactions, and protein-disease associations to achieve better predictive results.

## Data Availability Statement

The original contributions presented in the study are included in the article/supplementary material, further inquiries can be directed to the corresponding author.

## Author Contributions

XL conceived, designed and managed the study, analyzed the results, and revised the manuscript. YC conducted the experiments, analyzed the results, and wrote the manuscript. Both authors read and approved the final manuscript.

## Conflict of Interest

The authors declare that the research was conducted in the absence of any commercial or financial relationships that could be construed as a potential conflict of interest.

## Publisher’s Note

All claims expressed in this article are solely those of the authors and do not necessarily represent those of their affiliated organizations, or those of the publisher, the editors and the reviewers. Any product that may be evaluated in this article, or claim that may be made by its manufacturer, is not guaranteed or endorsed by the publisher.
